# Bipolar disorder: Construction and analysis of a joint diagnostic model using random forest and feedforward neural networks

**DOI:** 10.1016/j.ibneur.2024.07.007

**Published:** 2024-07-31

**Authors:** Ping Sun, Xiangwen Wang, Shenghai Wang, Xueyu Jia, Shunkang Feng, Jun Chen, Yiru Fang

**Affiliations:** a Qingdao Mental Health Center, Shandong 266034, China; b Clinical Research Center, Shanghai Mental Health Center, Shanghai Jiao Tong University School of Medicine, Shanghai 200030, China; c School of Mental Health, Research Institute of Mental Health,Jining Medical University, Shandong 272002, China; d Department of Psychiatry & Affective Disorders Center, Ruijin Hospital, Shanghai Jiao Tong University School of Medicine, Shanghai 200025, China; e Shanghai Key Laboratory of Psychotic Disorders, Shanghai 201108, China; f State Key Laboratory of Neuroscience, Shanghai Institue for Biological Sciences, CAS, Shanghai 200031, China; g Department of Medicine,Qingdao University, Shandong 266000, China

**Keywords:** Bipolar disorder, Machine learning, Neural networks, Diagnostic models

## Abstract

**Background:**

To construct a diagnostic model for Bipolar Disorder (BD) depressive phase using peripheral tissue RNA data from patients and combining Random Forest with Feedforward Neural Network methods.

**Methods:**

Datasets GSE23848, GSE39653, and GSE69486 were selected, and differential gene expression analysis was conducted using the limma package in R. Key genes from the differentially expressed genes were identified using the Random Forest method. These key genes' expression levels in each sample were used to train a Feedforward Neural Network model. Techniques like L1 regularization, early stopping, and dropout layers were employed to prevent model overfitting. Model performance was then validated, followed by GO, KEGG, and protein-protein interaction network analyses.

**Results:**

The final model was a Feedforward Neural Network with two hidden layers and two dropout layers, comprising 2345 trainable parameters. Model performance on the validation set, assessed through 1000 bootstrap resampling iterations, demonstrated a specificity of 0.769 (95 % CI 0.571–1.000), sensitivity of 0.818 (95 % CI 0.533–1.000), AUC value of 0.832 (95 % CI 0.642–0.979), and accuracy of 0.792 (95 % CI 0.625–0.958). Enrichment analysis of key genes indicated no significant enrichment in any known pathways.

**Conclusion:**

Key genes with biological significance were identified based on the decrease in Gini coefficient within the Random Forest model. The combined use of Random Forest and Feedforward Neural Network to establish a diagnostic model showed good classification performance in Bipolar Disorder.

## 1 Introduction

Bipolar Disorder (BD) is a complex neuropsychiatric disorder characterized by recurrent manic and depressive episodes. Despite its high prevalence worldwide, a deep understanding of its genetic and pathophysiological mechanisms continues to face numerous challenges (Grande et al., 2016). Meanwhile, the pathophysiological understanding and diagnostic processes for BD remain inadequate, often leading to delays in accurate diagnosis and appropriate management. Traditional diagnostic methods rely heavily on clinical observations and patient history, which are subjective and prone to misdiagnosis (Shen et al., 2018, Calvo et al., 2016).

Recent advancements in high-throughput sequencing technologies have opened new avenues for elucidating the pathogenesis of psychiatric disorders such as BD (Goodwin et al., 2016). However, in the diagnostic study of complex psychiatric diseases, research on other tissue samples is often more practical due to the general difficulty in obtaining brain tissue samples. Particularly, peripheral tissue RNA sequencing data has become an important sample for studying expression profiles and biomarker changes related to psychiatric diseases due to its ease of acquisition, non-invasiveness, and its ability to reflect, to some extent, the systemic changes and underlying pathophysiological processes characteristic of mental illnesses (Sullivan et al., 2006, Lee et al., 2020, Hess et al., 2020a).

With the continuous development of genomics technologies, the increasing throughput and read length of sequencing pose the challenge of how to eliminate biases caused by sample differences and background noise, and to select key biological features. The large scale, diversity, and high dimensionality of 'omics' data have driven the demand for more advanced technologies.

Random Forest, first proposed by Ho Tin Kam in 1995 (Ho, 1995), is a machine learning method used for classification, regression, and other tasks. It combines a large number of classification or regression trees, each dependent on the values of a randomly sampled vector, and with each tree in the forest having a similar distribution (Breiman, 2001). This design maintains high robustness and a lower risk of overfitting in situations involving multiple factors or nonlinear effects (Roy and Larocque, 2012), and can fit complex interactions in the data without prior knowledge (Wright et al., 2016). Furthermore, in its classification tasks, it can also rank the importance of variables (Liaw and Wiener, 2002a). This method has been widely applied in prognostic assessment (Wang et al., 2018), feature selection (Chen et al., 2020), and statistical testing methods (Zhang et al., 2022).

With the development of modern deep learning technologies, methods represented by neural network technology have gradually gained attention, increasingly demonstrating effectiveness in modeling subtle patterns and complex interactions in data (Wang et al., 2023, Liao et al., 2023, Zeng et al., 2023).

In this study, we utilized peripheral tissue RNA sequencing data from BD patients, combining random forest and neural network technologies, to explore their genetic changes from a machine learning perspective. This approach aims to reveal the genetic patterns of BD and attempts to construct a discriminative model, providing new insights for future diagnostic and therapeutic strategies.

## 2 Materials and methods

### 2.1 Data download and preprocessing

Using the GEOquery (Davis and Meltzer, 2007) package in R, data from the Gene Expression Omnibus (GEO) database of the National Center for Biotechnology Information (NCBI) were downloaded. This included the unstandardized expression matrices and clinical phenotype data for Illumina chip datasets GSE23848, GES69486, and GSE39653, as shown in Table 1. In the initial phase of data processing, the datasets were subjected to background correction and normalization using the neqc method (Shi et al., 2010), encapsulated within the limma (Ritchie et al., 2015) package. Subsequently, probes were re-annotated against the Gencode_v44 (Frankish et al., 2019) reference genome using the Rsubread (Liao et al., 2019) package,During the mapping process from chip probe IDs to gene names (Gene Symbol), cases where a single gene symbol corresponded to multiple probes were eliminated. For scenarios where multiple probes corresponded to a single gene symbol, the average expression of these probes was used to represent the expression level of that gene.

**Table 1:** Basic Information on the Gene Sets Used in the Study.

Dataset	Sample Size	Tissue Type	Sequencing Platform
GSE23848	35 (15 Healthy, 20 Bipolar)	Peripheral Whole Blood	Sentrix Human−6 v2 Expression BeadChip
GSE39653	53 (24 Healthy, 8 Bipolar)	Peripheral Blood Mononuclear Cells	Illumina HumanHT−12 V4.0 expression beadchip
GSE69486	12 (5 Healthy, 10 Bipolar Disorder)	Dermal Fibroblasts	Illumina HumanHT−12 V4.0 expression beadchip

### 2.2 Differential expression and enrichment analysis

Differential expression analysis was performed on each of the three datasets using the limma package in R. Limma utilizes a classic Bayesian method to identify differentially expressed genes (DEGs) and provides the adjusted p-values (adj.p) for each gene. Subsequently, the Robust rank aggregation (RAA) method (Kolde et al., 2012) was used to construct lists of up-regulated and down-regulated genes based on adj.p in each of the three datasets, retaining genes with a Score < 0.05 as DEGs. Furthermore, the R package clusterProfiler (Wu et al., 2021) was employed for Gene Ontology (GO) analysis and Kyoto Encyclopedia of Genes and Genomes (KEGG) enrichment analysis of the DEGs, to identify significantly enriched GO terms (P < 0.05, Q < 0.05) and KEGG pathways (P < 0.05, Q < 0.05).

### 2.3 Feature gene selection

Initially, common genes across the three datasets were identified and their raw expression levels merged. Batch effects were then removed using the ComBat method from the SVA package (Daniel et al., 2022), ensuring data consistency and comparability.

Subsequently, DEGs were selected and input into a random forest model for feature selection. To determine the two key parameters of the random forest model, mtry (the number of variables considered at each tree split) and ntree (the number of trees), a grid-based parameter search scheme was utilized. The range for mtry was set from 1 to the number of features in the training data, while ntree was fixed at five levels(100,200,500,1000,2000). Stratified 10-fold cross-validation was employed to more accurately assess model performance. Parallel computing was introduced to expedite computation, with the model trained on training subsets and predictions made on validation subsets under each parameter combination, thereby calculating the error rate for each fold. The optimal parameter combination was determined by summarizing the average error rate for all parameter combinations.

A random forest model was built using the optimal parameters to classify all samples. The importance of the output results was measured in terms of reduced accuracy and decreased mean squared error (Gini coefficient method). To ensure robustness, the importance measurement process included 10 rounds of 5-fold cross-validation, with each fold containing samples of both healthy controls and bipolar disorder patients. The decrease in Gini coefficient value contributed by each gene in each iteration was accumulated and ranked. Finally, the top 50 genes in terms of importance were selected as key genes for subsequent model construction. The software package pheatmap (Kolde, 2019) was used to reclassify the 50 important genes through unsupervised hierarchical clustering and to generate a heatmap. The random forest model was implemented using the randomForest package (Liaw and Wiener, 2002b), with parallel processing and cross-validation facilitated by the caret (Kuhn, 2008)and foreach packages.

### 2.4 Neural network construction for disease classification model

Following Z-standardization of the expression levels of key genes, 70 % of the samples were randomly selected as the training set, with the remaining 30 % as the test set. A multilayer perceptron model was constructed using the Keras framework, comprising an input layer, an output layer, two fully connected layers, and two dropout layers. To identify the best parameters for the model, Bayesian optimization was utilized to select the number of nodes in each layer, The objective function is defined as the validation loss of the MLP model. The search space for the number of nodes in each hidden layer is set from 32 to 256,seeking the optimal parameter combination to maximize model performance. The 50 identified key genes were used as model inputs. The AUC classification performance was validated using the pROC software package, and the performance interval was computed through 1000 resampling iterations using the Bootstrap method.

## 3 Results

### 3.1 Differential expression

**Fig. 1:**
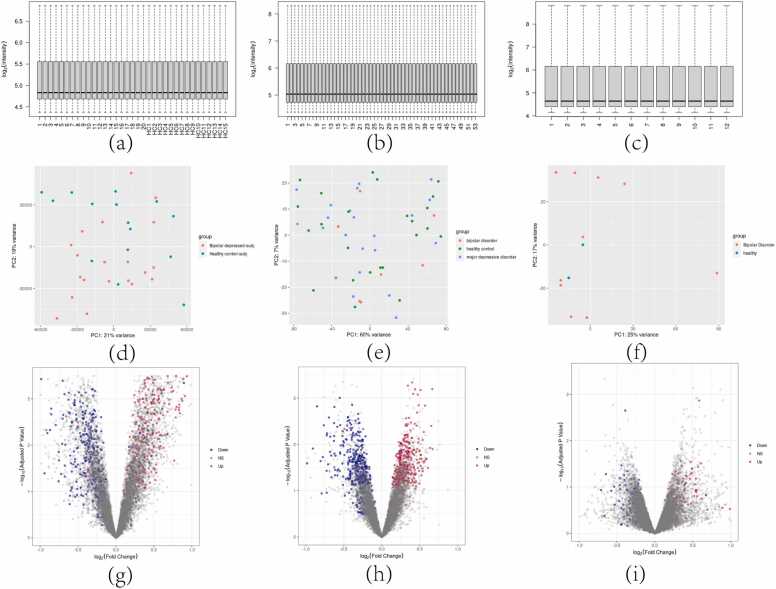
"Post-Processing Data Visualization". (a)(b)(c)Post-background correction and normalization expression levels for datasets GSE23848, GSE39653, and GES69486, respectively.(d)(e)(f): PCA plots following background correction and normalization for the same datasets.(h)(i)(g): Volcano plots of differentially expressed genes identified by RRA for each of the three datasets.

Differential expression analysis, utilizing re-annotated and background-corrected and normalized probe data, combined with the Robust Rank Aggregation (RRA) method, identified a total of 1227 differentially expressed genes (DEGs). Specifically, 576 genes were significantly upregulated, while 651 genes were significantly downregulated across all three datasets. The re-annotation results are shown in Table 2. The relevant pretreatment situation and the volcano map of key RRA genes are shown in Fig. 1.

**Table 2:** Reannotation Results.

Sequencing Platform	Platform ID	Total_reads	Mapped	Uniquely_mapped_reads
Illumina HumanHT−12 V4.0 expression beadchip	GPL10558	48107	41522	14667
Sentrix Human−6 v2 Expression BeadChip	GPL6106	48701	33628	12876

### 3.2 Enrichment analysis

**Figure 2:**
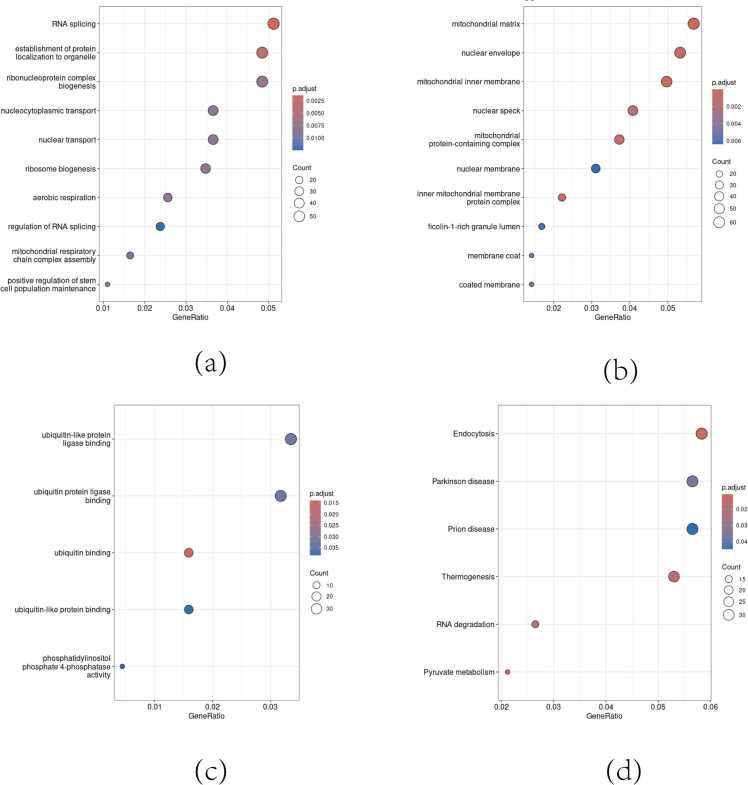
"GO and KEGG Enrichment Analysis" (a): GO enrichment in Biological Process (BP),(b): GO enrichment in Cellular Component (CC), (c): GO enrichment in Molecular Function (MF), (d): KEGG pathway enrichment results - bubble size for number of genes, color depth for enrichment significance.

GO enrichment analysis was conducted on 1227 significant DEGs using the clus-terProfiler software package. The significance threshold was set using the Benja-mini-Hochberg correction method at P < 0.05 and Q < 0.05. The analysis highlighted a significant over-representation of biological processes such as RNA splicing and nucleocytoplasmic transport, indicating regulatory alterations in gene expression. Cellular components associated with mitochondrial structures, such as the mitochondrial matrix and inner membrane, were notably enriched, suggesting dysregulated energy metabolism in the disorder. Molecular function enrichment pointed to ubiquitin-related protein binding, implying changes in protein modification processes. Furthermore, pathway analysis shed light on systemic biological themes, revealing the involvement of endocytosis and thermogenesis pathways, which may have implications for the pathophysiology of bipolar disorder beyond the central nervous system. The bubble diagram for enrichment analysis is shown in Figure 2.

### 3.3 Random forest feature extraction

Initially, batch effects in the DEGs obtained through the RRA method across the three datasets were removed using the ComBat function in the sva package. After removing batch effects, gene expressions were standardized using z-normalization to ensure uniform comparison standards in the random forest model.

Through grid-based parameter optimization, we fine-tuned two critical parameters of the random forest model: ntree (number of trees in the forest) and mtry (number of variables randomly selected at each node split). The optimal parameter combination was determined as ntree = 300 and mtry = 41, as it exhibited the lowest average error rate, balancing model complexity.

**Figure 3:**
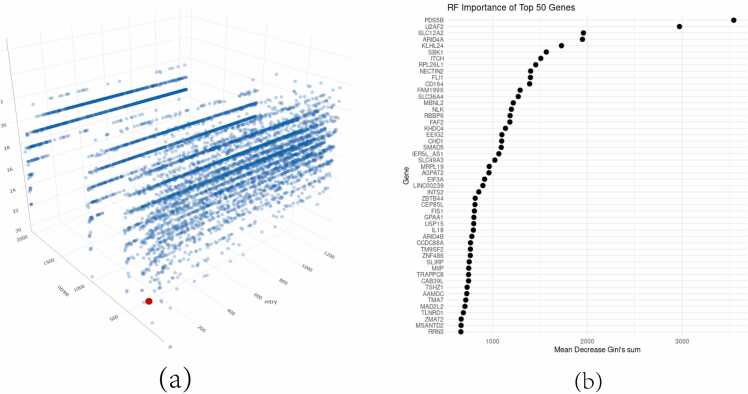
"Random Forest Optimization and Key Features". (a): The 3D scatter plot represents the results of a grid search optimization for the random forest parameters, plotting 'mtry' on the X-axis, 'ntree' on the Y-axis, and the average error rate on the Z-axis. The red point denotes the selected optimal parameters that balance model complexity with error rate. (b): Top 50 key features selected based on the decrease in Gini coefficient, depicted in a relevant format (like a bar chart or ranked list).

**Fig. 4:**
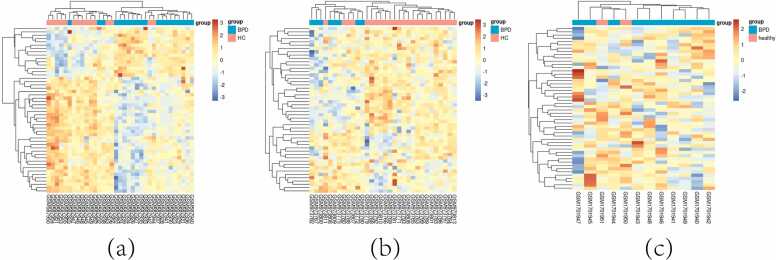
"Key Gene Expression Heatmaps". (a): Cluster heatmap showing the top 50 genes selected by the Random Forest algorithm in dataset GSE23848.(b): Cluster heatmap for the top 50 genes in dataset GSE39653 as identified by the Random Forest method.(c): Cluster heatmap depicting the top 50 genes from dataset GES69486, selected using Random Forest analysis.

Upon establishing the optimal parameter combination, we conducted 10 rounds of 10-fold cross-validation on all DEGs, recording the normalized decrease in Gini values for each gene in every iteration. This metric helped assess the importance of each gene from the perspectives of reducing accuracy and minimizing mean squared error. Based on this metric, the top 50 genes were selected. These 50 genes demonstrated significant importance in our random forest model, leading us to believe they are key genes closely related to the disease. The model parameters and the genes in focus are shown in Figure 3.The heatmaps of the expression of these genes in the three datasets are shown in Fig. 4.

### 3.4 Neural network construction for disease classification model

**Fig. 5:**
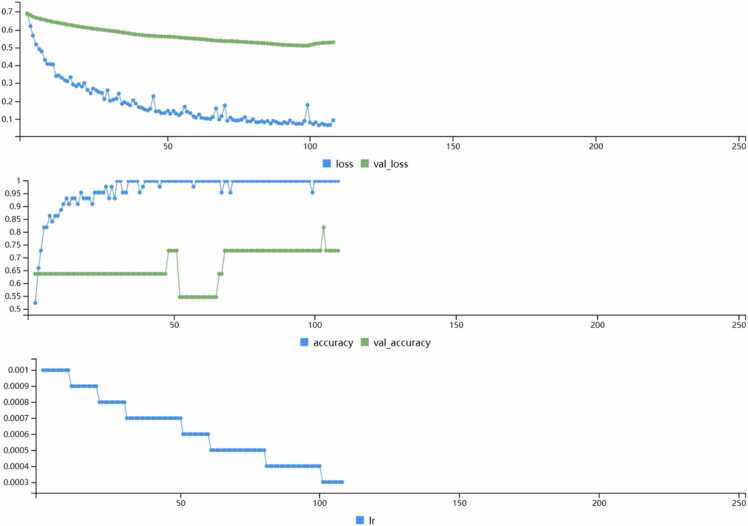
"Neural Network Training Metrics". Loss and Validation Loss (Top Graph): The first graph shows the loss on the training dataset (blue line) and the validation dataset (green line) as the model trains over epochs. Initially, both training and validation loss decrease, indicating that the model is learning. However, the validation loss stabilizes after a certain number of epochs, which might suggest the point of convergence.Accuracy and Validation Accuracy (Middle Graph): The second graph tracks the accuracy on the training set (blue line) and the validation set (green line). Both accuracies improve rapidly in the initial epochs and then plateau, with training accuracy remaining consistently higher than validation accuracy. The spikes in validation accuracy indicate moments of high generalization performance.Learning Rate (Bottom Graph): The third graph illustrates the learning rate (lr) of the model over epochs. It shows a step-wise decay in the learning rate, which is a common technique to help the model converge by taking smaller steps in the optimization landscape as training progresses.

Prior to training the neural network, the expression levels of 50 key genes were Z-standardized. 70 % of the samples were used as the training set, with the remainder serving as the validation set. A multilayer perceptron (MLP) model, a specific instance of a feedforward neural network (FNN), was then constructed. This model included an output layer, two fully conn ected layers, and two dropout layers, with a dropout layer following each fully connected layer, randomly deactivating 20 % of neurons (Srivastava et al., 2014). ReLU was used as the activation function in each fully connected layer, setting all negative inputs to zero while leaving positive inputs unchanged, which speeds up convergence in training deep neural networks and helps alleviate the vanishing gradient problem (Nair and Hinton, 2010). L1 regularization was added in each fully connected layer to prevent overfitting. An early stopping strategy was employed during training to prevent the model from learning noise in the dataset, terminating training when there was no decrease in validation set loss (val_loss) for 10 consecutive trainings. The model underwent 108 training iterations, resulting in a model with 2345 parameters. The model's performance on the validation set was assessed through 1000 bootstrap resampling iterations, calculating a 95 % confidence interval. The model demonstrated a specificity of 0.769 (95 % CI 0.571–1.000), sensitivity of 0.818 (95 % CI 0.533–1.000), AUC of 0.832 (95 % CI 0.642–0.979), and accuracy of 0.792 (95 % CI 0.625–0.958) on the test set, indicating good fit to the dataset and high predictive value. The model training process is shown in Fig. 5.

### 3.5 Exploration of biological functions of key genes

**Fig. 6:**
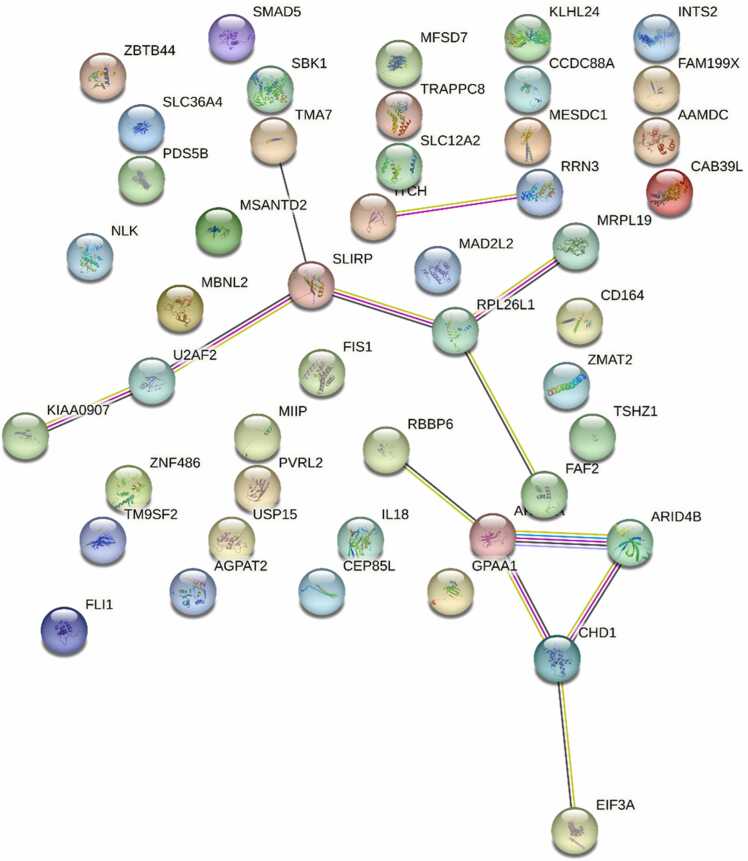
"PPI Network Map". A network map illustrating the protein-protein interactions of the 50 key genes.

To explore the biological functions of the key genes, GO, KEGG enrichment analysis, and Protein-Protein Interaction (PPI) network construction were performed. No significant GO terms or KEGG pathways were enriched. A PPI network was constructed using medium confidence, resulting in a network structure with 47 nodes, 12 edges, and an average node degree of 0.511. The PPI graph is shown in Fig. 6.

## 4 Discussion

Bipolar Disorder is a complex polygenic disorder, and its key genetic determinants are yet to be clearly defined (Craddock and Sklar, 2013). With advancements in omics technologies and the reduction of sequencing costs, exploring the pathophysiological processes or early diagnosis of BD at the genetic expression level has become a possibility (Akula et al., 2014, Akbarian et al., 2015). However, omics data often present challenges in managing high-dimensional and large datasets. The effective utilization of these data has been extensively explored and studied in non-psychiatric fields (Li et al., 2018, Jagga and Gupta, 2015).

In this study, the Random Forest algorithm, an ensemble machine learning method that builds multiple decision trees and outputs the mode of the categories (for classification problems) or mean prediction (for regression problems), was employed (Breiman, 2001). This ensemble learning method is particularly suitable for handling high-dimensional data because it randomly selects a subset of features at each node of the tree, providing robustness against overfitting and offering a measure of feature importance, which can be used to identify key variables (Chen and Ishwaran, 2012). This makes the Random Forest algorithm a powerful tool for feature selection in high-dimensional omics data (Barnes et al., 2005, Strobl et al., 2007). Recent studies have shown that, compared to popular algorithms like LASSO, Ridge regression, and Elastic Net, it performs better in accuracy and in modeling complex interactions (Boulesteix et al., 2012, Tolosi and Lengauer, 2011). This can better capture the complex polygenic interaction mechanisms inherent in BD.

Subsequently, a Multilayer Perceptron (MLP) model, a type of artificial neural network, was constructed for disease diagnosis. MLPs, with multiple layers and neurons, offer high flexibility and, through iterative training, enable the model to learn subtle and complex interaction patterns that may exist in diseases (Min et al., 2017). To mitigate overfitting, common in deep learning models, methods like L1 regularization, early stopping, and learning rate decay were used. Model performance validation on the validation set demonstrated strong classification capabilities, though there was a decline compared to the training dataset. This could be attributed to differences between the two datasets or potential overfitting of the model to the training data, leading to reduced performance on new, unseen data.

Our study introduces a pioneering diagnostic model for BD that leverages peripheral tissue RNA data. The multilayer perceptron model demonstrated strong classification performance on the validation set, with a specificity of 0.769 (95 % CI 0.571–1.000), sensitivity of 0.818 (95 % CI 0.533–1.000), AUC value of 0.832 (95 % CI 0.642–0.979), and accuracy of 0.792 (95 % CI 0.625–0.958). These metrics indicate that the model effectively distinguishes between BD patients and healthy individuals.

The implications of these performance metrics are significant given the complex genetic background of BD. The high sensitivity and AUC values suggest that the model successfully captures subtle genetic variations and interactions characteristic of BD. However, the lower specificity highlights potential challenges in distinguishing BD from other psychiatric disorders with overlapping genetic profiles.

This novel approach promises to enhance diagnostic accuracy and significantly contribute to our understanding of BD’s pathophysiology. Compared to previous studies utilizing peripheral blood gene expression profiles, the incorporation of machine learning techniques in our model has greatly enhanced its capabilities (Hess et al., 2020b).

To further explore the biological functions of key genes, enrichment analysis and PPI network modeling of the 50 genes were performed. Enrichment analysis did not yield significant GO terms or KEGG pathways, suggesting that the key genes identified through machine learning may be involved in multiple pathways and biological processes without significant enrichment in any one area. This observation highlights the complexity and heterogeneity of the genetic architecture of BD. This complexity is supported by results from various whole-genome sequencing studies. For example, research using data from the Old Order Amish families showed that although linkage analyses among BD patients identified several linkage peaks, further investigations found no evidence of specific risk loci or common pathways (Georgi et al., 2014).Similarly, a study from India also arrived at comparable conclusions (Kumar et al., 2000).These findings emphasize the multifaceted genetic underpinnings of BD, which may not converge on a common set of pathways or loci.

The Protein-Protein Interaction (PPI) network results indicated some biological interconnectivity among proteins encoded by key genes. However, the network's structure was relatively sparse, suggesting these genes are key in various biological functions and are not concentrated in specific pathways or are distantly related.

This could be due to the Random Forest method capturing subtle associations between genes. These subtle associations might have significant impacts on individual gene expression changes or disease states but may not yet be fully explored or understood in current biological research.

It is worth noting that, although there was no significant enrichment of biological pathways or functions among the key genes, investigating these genes individually suggests their potential involvement in the pathogenesis of BD. According to GWAS using data from the UK biobank, a total of 36 missense mutations in the ARID4A gene showed that the allele frequencies were different in the control and mental disorder cases. Thirteen (36.1 %) loci were associated with bipolar and major depression conditions (5418 cases and 68724 controls) (Ren et al., 2022). Furthermore, among the top five genes ranked by importance, U2AF2 (Kittock et al., 2023) and SLC12A2 (Lam et al., 2023, Panichareon et al., 2012) have also been shown to play a role in the pathogenesis of psychiatric disorders.

The integration of Random Forest and Feedforward Neural Networks in our study has highlighted the potential of machine learning in uncovering subtle genetic markers linked to BD. The performance metrics of our model, particularly the high sensitivity and AUC value, have significant clinical implications in the genetic background of BD. BD is a highly genetically heterogeneous psychiatric disorder involving multiple genes and complex molecular mechanisms. Our model's high sensitivity and AUC value indicate that the gene expression patterns derived from peripheral blood RNA sequencing data effectively capture the genetic characteristics of BD. These gene expression changes may reflect systemic biological processes in BD patients, including neurotransmitter metabolism, cell signaling, and gene regulation.

While our key genes identified did not align with known pathways through enrichment analysis, they offer a novel direction for research. Future studies could focus on longitudinal data to monitor changes in these genes' expressions across BD episodes, integrate larger omics datasets for a more comprehensive genetic network analysis, and conduct functional validation through gene manipulation experiments. Furthermore, the clinical relevance of these genes could be evaluated in predictive diagnostics and treatment response trials, fostering a move towards personalized medicine. Lastly, comparing these findings across other psychiatric disorders could elucidate common molecular mechanisms, providing insights into the complex nature of psychiatric comorbidities. As a next step, we plan to collect additional samples for analysis to further validate our findings. These potential research directions will not only validate our current findings but also expand our understanding, supporting the evolution of precision psychiatry that incorporates genetic and biomarker data into clinical practice.

Despite providing new insights into the molecular mechanisms of BD, this study has limitations. Firstly, our data reliance on public databases may lack in-depth understanding of samples and not fully eliminate confounding factors like gender, age, and medication treatment. Secondly, the limited sample size may affect statistical power. We emphasize that while our model shows promise in distinguishing between BD patients and healthy individuals, its clinical applicability requires further validation in larger, independent cohorts and consideration of potential confounding factors. Lastly, by focusing primarily on genetic factors, the study may overlook the role of environmental factors, necessitating validation in functional experiments.

## 5 Conclusions

In this study, datasets GSE23848, GES69486, and GSE39653 were subjected to background correction and normalization to correct for systematic variations that might not be attributable to biological differences. Differential expression analysis identified 1227 significant differentially expressed genes (DEGs), pinpointing potential genes associated with BD. An initial exploration of the biological functions of these DEGs was conducted through Gene Ontology (GO) and KEGG analysis. The analysis highlighted a significant over-representation of biological processes such as RNA splicing and nucleocytoplasmic transport, indicating regulatory alterations in gene expression. Cellular components associated with mitochondrial structures, such as the mitochondrial matrix and inner membrane, were notably enriched, suggesting dysregulated energy metabolism in the disorder. Molecular function enrichment pointed to ubiquitin-related protein binding, implying changes in protein modification processes. Furthermore, pathway analysis shed light on systemic biological themes, revealing the involvement of endocytosis and thermogenesis pathways, which may have implications for the pathophysiology of BD beyond the central nervous system.

Subsequently, 50 genes closely associated with the disease were selected as key genes using the Random Forest algorithm. Based on the expression patterns of these 50 key genes, a Multilayer Perceptron model was established. The model's performance on the test set, with a specificity of 0.769 (95 % CI 0.571–1.000), sensity of 0.818 (95 % CI 0.533–1.000), AUC value of 0.832 (95 % CI 0.642–0.979), and accuracy of 0.792 (95 % CI 0.625–0.958), demonstrates its capability to distinguish between patients and healthy individuals to a certain extent.

Finally, the diagnostic genes identified by the machine learning algorithm were analyzed through enrichment analysis and Protein-Protein Interaction (PPI) network construction to compare and contrast with current biological research consensus. This approach provided insights into the similarities and differences between the machine-learned diagnostic genes and those established in the field of biological research. This integrative method underscores the potential of combining advanced computational approaches with traditional biological research to enhance our understanding of complex disorders like BD.

## Ethics approval and consent to participate

This study involved the analysis of publicly available data obtained from the Gene Expression Omnibus (GEO) database. As this research did not involve direct data collection from human participants or animals, ethical approval and consent to participate are not applicable. However, all procedures followed were in accordance with the standards and guidelines of the GEO database.

## Funding

The work was supported by the 10.13039/501100001809National Natural Science Foundation of China(81930033) and the Shanghai Municipal Science and Technology Major Project(2018SHZDZX05).

## CRediT authorship contribution statement

**Shenghai Wang:** Conceptualization. **Yiru Fang:** Writing – review & editing, Supervision. **Jun Chen:** Writing – review & editing, Supervision. **Shunkang Feng:** Visualization, Software. **Xueyu Jia:** Data curation. **Xiangwen Wang:** Software, Methodology, Data curation, Conceptualization. **Ping Sun:** Writing – original draft, Project administration, Conceptualization.

## Declaration of Generative AI and AI-assisted technologies in the writing process

During the preparation of this work, the author(s) utilized OpenAI's ChatGPT to improve the language and grammar, thereby enhancing the readability of the article. Following the use of this AI tool, the author(s) reviewed and edited the content as necessary and take(s) full responsibility for the content of the publication.

## Declaration of Competing Interest

The authors declare that they have no competing interests

## Data Availability

The datasets generated and/or analysed during the current study are available in the Gene Expression Omnibus(GEO) repository, http://www.ncbi.nlm.nih.gov/geo/query/acc.cgi?acc=gse23848, http://www.ncbi.nlm.nih.gov/geo/query/acc.cgi?acc=gse39653, http://www.ncbi.nlm.nih.gov/geo/query/acc.cgi?acc=gse69486
